# Cytoplasmic delivery of siRNA using human-derived membrane penetration-enhancing peptide

**DOI:** 10.1186/s12951-022-01667-4

**Published:** 2022-10-27

**Authors:** Momoko Nakamura, Kei Fujiwara, Nobuhide Doi

**Affiliations:** Department of Biosciences and Informatics, Keio University, 3-14-1 Hiyoshi, Yokohama, 223-8522 Japan

**Keywords:** Argonaute 2, Cell-penetrating peptide, Endosomal escape, Fusogenic peptide, Gene knockdown

## Abstract

**Background:**

Although protein-based methods using cell-penetrating peptides such as TAT have been expected to provide an alternative approach to siRNA delivery, the low efficiency of endosomal escape of siRNA/protein complexes taken up into cells by endocytosis remains a problem. Here, to overcome this problem, we adopted the membrane penetration-enhancing peptide S19 from human syncytin 1 previously identified in our laboratory.

**Results:**

We prepared fusion proteins in which the S19 and TAT peptides were fused to the viral RNA-binding domains (RBDs) as carrier proteins, added the RBD-S19-TAT/siRNA complex to human cultured cells, and investigated the cytoplasmic delivery of the complex and the knockdown efficiency of target genes. We found that the intracellular uptake of the RBD-S19-TAT/siRNA complex was increased compared to that of the RBD-TAT/siRNA complex, and the expression level of the target mRNA was decreased. Because siRNA must dissociate from RBD and bind to Argonaute 2 (Ago2) to form the RNA-induced silencing complex (RISC) after the protein/siRNA complex is delivered into the cytoplasm, a dilemma arises: stronger binding between RBD and siRNA increases intracellular uptake but makes RISC formation more difficult. Thus, we next prepared fusion proteins in which the S19 and TAT peptides were fused with Ago2 instead of RBD and found that the efficiencies of siRNA delivery and knockdown obtained using TAT-S19-Ago2 were higher than those using TAT-Ago2. In addition, we found that the smallest RISC delivery induced faster knockdown than traditional siRNA lipofection, probably due to the decreased time required for RISC formation in the cytoplasm.

**Conclusion:**

These results indicated that S19 and TAT-fused siRNA-binding proteins, especially Ago2, should be useful for the rapid and efficient delivery of siRNA without the addition of any endosome-disrupting agent.

**Supplementary Information:**

The online version contains supplementary material available at 10.1186/s12951-022-01667-4.

## Background

In recent years, membrane-active peptides such as cell-penetrating peptides (CPPs) have been widely used for the intracellular delivery of various biomacromolecules, such as proteins and nucleic acids, as well as nanomaterials for biomedical and nanobiotechnological applications [1–10]. The first and most commonly used CPP is a cationic peptide TAT derived from HIV1 that binds to the cell surface by electrostatic interaction and is taken up into cells by endocytosis [11, 12]. Although there is concern about the low efficiency of endosomal escape after uptake into cells [1, 2, 9], several approaches have been shown to enhance the efficiency of endosomal escape of the TAT peptide [13–15]. We previously found that the 19 amino-acid fusogenic peptide named S19 from human syncytin 1, a protein involved in cell membrane fusion in placenta formation, enhances the efficiency of the intracellular delivery of TAT-fused proteins [16]. In this study, we applied the membrane penetration-enhancing peptide S19 to the cytoplasmic delivery of small interfering RNA (siRNA).

siRNA causes RNA interference (RNAi), which mediates sequence-specific cleavage of target mRNAs. Long precursor double-stranded (ds) RNA is processed by Dicer2 into a 21–23 nt small RNA called the siRNA duplex, followed by the formation of RNA-induced silencing complex (RISC), which cleaves a target mRNA with a complementary sequence region. Although many proteins are known to be involved in RISC formation, the complex of siRNA and Argonaute 2 (Ago2), which acts as the core of RISC, is defined as the smallest RISC [17]. For knockdown experiments on a target gene of interest in vitro, lipofection reagents (*e.g*., Lipofectamine) have been widely used for the transfection of siRNA into cells, although this approach exhibits substantial cytotoxicity [18]. For therapeutic applications, lipid nanoparticles (LNPs) or GalNAc conjugates have been successfully used in FDA-approved siRNA therapeutics, although they have some limitations, such as accumulation in the liver [19, 20]. Thus, a variety of other siRNA delivery carriers have been reported; *e.g*., the lipid [21], peptide [22, 23], polymer [24], and inorganic [25] nanoparticles [26] and nanogels [27].

As another simple approach to siRNA delivery, protein-based methods have been proposed by several groups [28–32]. They combined cell-surface binders such as TAT with RNA-binding domains (RBDs) that bind to siRNA in a sequence-independent manner. In comparison with LNPs, the protein-based small delivery vehicles have the advantage of higher tissue permeability [33]. In comparison with GalNAc conjugates, siRNA embedded in RBD can be protected against degradation by nucleases [29]. However, the problem remains that the efficiency of endosomal escape of the siRNA/protein complex taken up into cells by endocytosis is low. To overcome this problem, we fused the membrane penetration-enhancing peptide S19 and TAT with RBD and investigated whether the intracellular uptake and knockdown activity of siRNA were improved. Furthermore, we fused S19 and TAT with Ago2 instead of RBD and tested the direct delivery of the smallest RISC into cells to build a simpler and more efficient delivery system.

## Results

### Application of the S19-TAT peptide to siRNA delivery using the viral RNA-binding domain

**Fig. 1:**
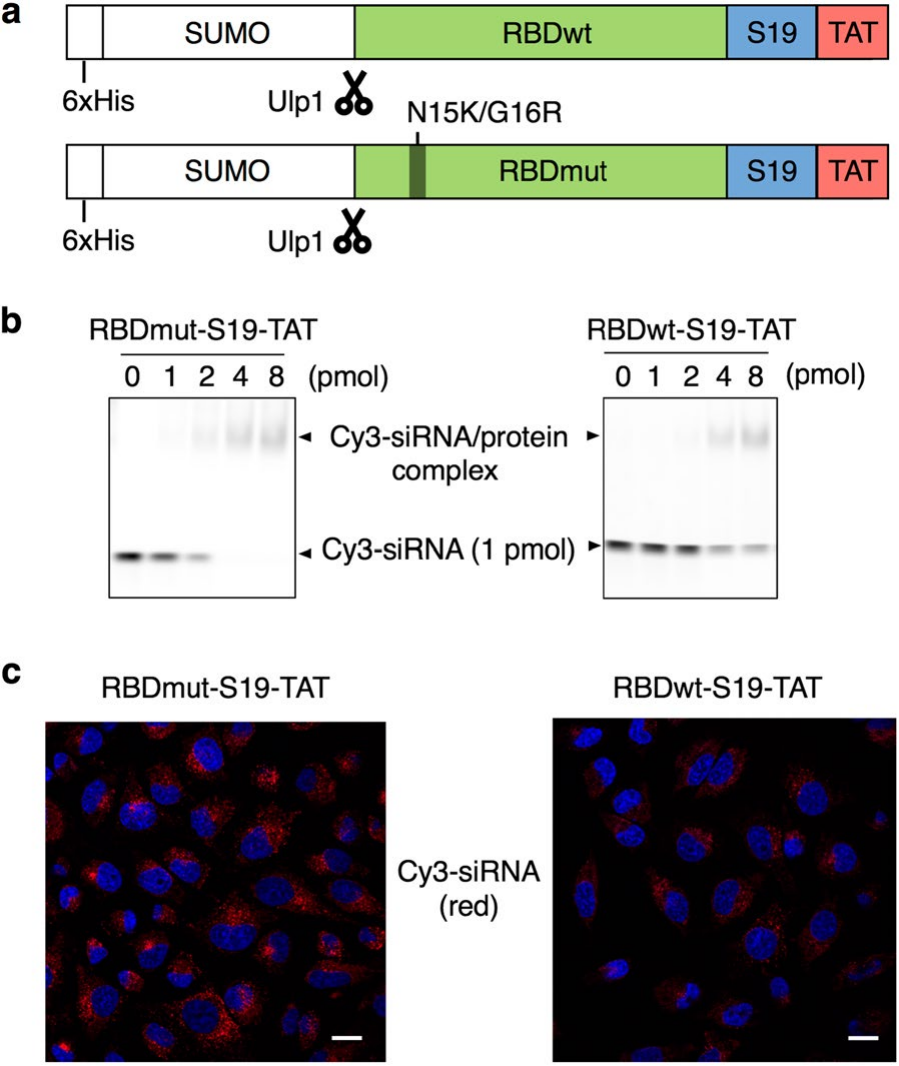
Intracellular delivery of the siRNA/RBD-S19-TAT complex. **a** DNA constructs for RBD-S19-TAT fusion proteins. **b** Confirmation of binding between the RBD-S19-TAT fusion protein and Cy3-labelled siRNA by electrophoretic mobility shift assay. **c** Confocal images of HeLa cells treated with the complex of RBD-S19-TAT protein (400 nM) and Cy3-labelled siRNA (100 nM) (red) for 1 h. Nuclei were stained with Hoechst 33,342 (blue). Scale bars, 20 μm

As the siRNA carrier protein, we chose RBD from the 19-kDa Tombsvirus protein with several mutations previously reported by Yang et al. [32]: they first introduced two mutations, C134S and C160A, into RBD to prevent uncontrolled crosslinking; here, we called this mutant RBDwt and the high-affinity mutant with the additional N15K and G16R mutations RBDmut (Fig. 1a). Whereas Yang et al. combined the pore-forming protein perfringolysin O as an endosome-disrupting agent *in trans* with the RBD/siRNA complex [32], we fused the membrane penetration-enhancing peptide S19 and TAT *in cis* with the RBD. By using the SLIC method [34], we constructed two plasmids based on the pET15 vector containing the T7 promoter for *E. coli* expression of RBDwt-S19-TAT and RBDmut-S19-TAT fusion proteins with the N-terminal 6 × His-SUMO-tag for affinity purification and Ulp1 cleavage [35] (Fig. 1a). We confirmed the expression and purification of these proteins by SDS–PAGE (Additional file 1: Fig. S1).

Then, we confirmed the binding of the purified RBD-S19-TAT proteins with Cy3-labelled siRNA by a gel shift assay. As shown in Fig. 1b, we observed a mobility shift of the Cy3-siRNA bands when more than 2 pmol of RBDmut-S19-TAT was added to 1 pmol of Cy3-labelled siRNA and when more than 4 pmol of RBDwt-S19-TAT was added to 1 pmol of siRNA, indicating that RBDmut has higher affinity for siRNA than RBDwt, as expected. In addition, we evaluated whether Cy3-siRNA bound to the RBD-S19-TAT protein is protected from nuclease degradation. We exposed the Cy3-siRNA or Cy3-siRNA/RBD-S19-TAT protein complex to bovine serum and determined the residual amount of Cy3-siRNA by native PAGE. After 3 h of bovine serum exposure, more than 80% of the siRNA was degraded in the absence of the RBD-S19-TAT protein, while most siRNA remained in the presence of the carrier protein (Additional file 1: Fig. S2).

**Fig. 2:**
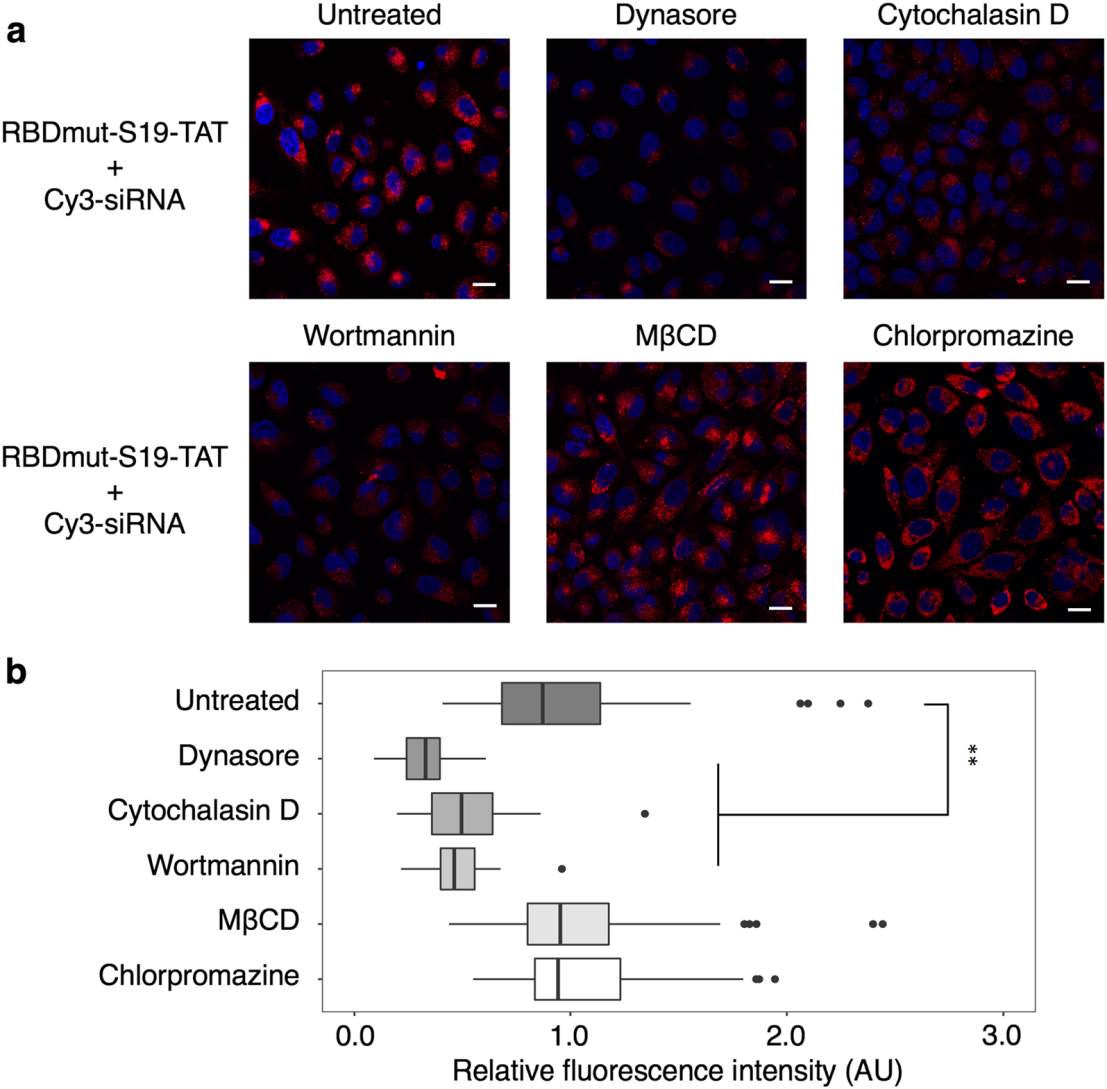
Effect of endocytosis inhibitors on the intracellular delivery of the complex of RBDmut-S19-TAT protein with Cy3-labelled siRNA. **a** Confocal images of HeLa cells treated with the complex of 400 nM RBDmut-S19-TAT and 100 nM Cy3-labelled siRNA (Red) in the absence (Untreated) or the presence of 80 μM dynasore, 1 μM cytochalasin D, 100 nM wortmannin, 1 mM MβCD or 50 μM chlorpromazine for 1 h and observed on live cells. Nuclei were stained with Hoechst 33,342 (blue). Scale bars, 20 μm. **b** ROI was taken for each cell and the average fluorescence intensity of Cy3 was quantified. N = 52, 38, 38, 24, 41 or 34; means ± SD; **p < 0.01

Next, we confirmed the intracellular uptake of the complex of Cy3-labelled siRNA with the RBD-S19-TAT protein into HeLa cells with a laser scanning confocal microscope (LSCM). When the RBDmut-S19-TAT complex with Cy3-siRNA was added to HeLa cells, Cy3 fluorescence was observed in the cells (Fig. 1c), whereas very little fluorescence was observed when siRNA alone or the siRNA complex with the RBDmut-TAT protein was added (Additional file 1: Fig. S3). This result verified that the S19 peptide promoted the intracellular delivery of the siRNA/protein complex. We also confirmed the uptake pathway of the siRNA/RBDmut-S19-TAT complex by using various endocytosis inhibitors. TAT-fusion proteins have previously been shown to bind to heparan sulfate proteoglycans on the cell surface and to be internalized by lipid raft-dependent macropinocytosis [13]. As a result, the intracellular fluorescence of Cy3-siRNA was suppressed in the presence of dynasore, cytochalasin D or wortmannin, all of which inhibit macropinocytosis, while Cy3 fluorescence was maintained in the presence of methyl-β-cyclodextrin (MβCD), which inhibits caveolin-dependent endocytosis, or chlorpromazine, which inhibits clathrin-dependent endocytosis (Fig. 2). Thus, the uptake pathway of the siRNA/RBDmut-S19-TAT complex was confirmed by TAT-mediated macropinocytosis. These results are consistent with a previous study on the intracellular delivery of eGFP-S19-TAT proteins [16]. Finally, we compared the intracellular uptake of Cy3-siRNA using RBDmut-S19-TAT and that using RBDwt-S19-TAT. As expected, when using RBDmut, which has a higher affinity for siRNA, more siRNA was taken up than when using RBDwt, which has a lower affinity (Fig. 1c).

**Fig. 3:**
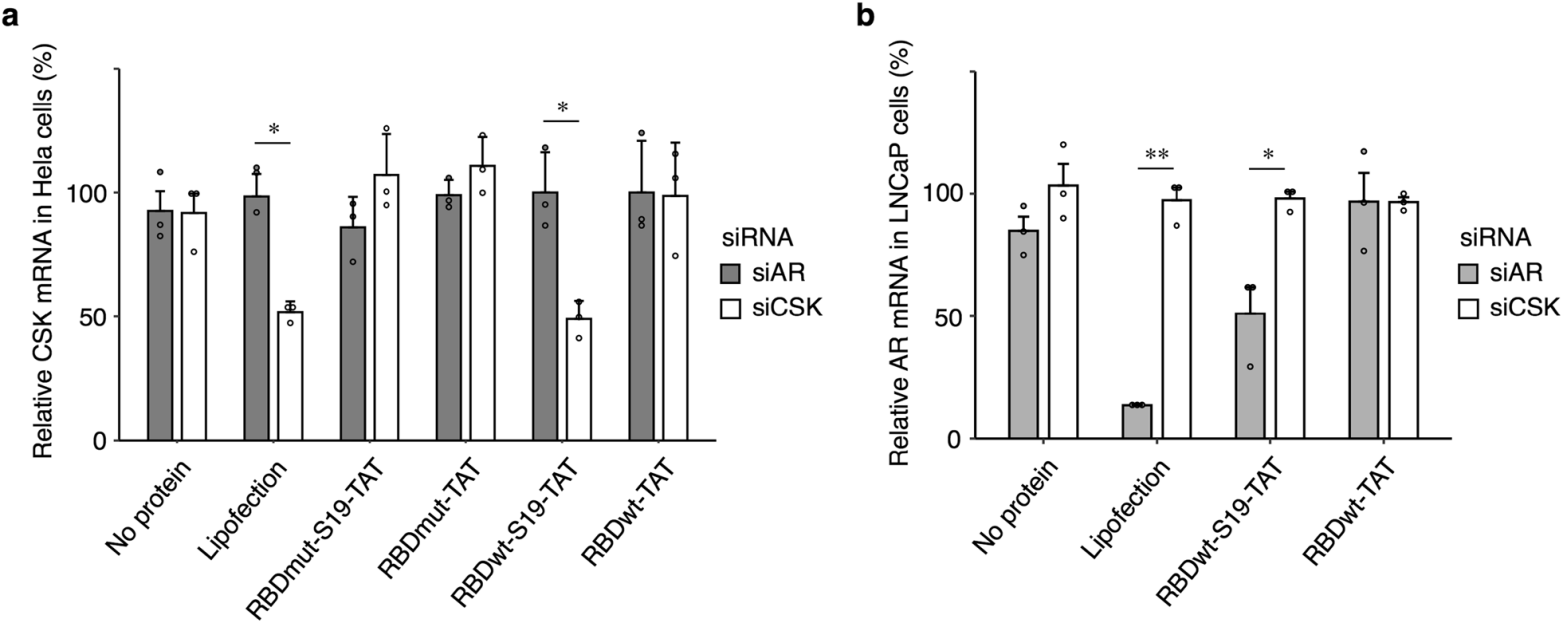
Gene knockdown with the RBD-S19-TAT protein/siRNA complex. **a** HeLa cells or **b** LNCaP cells were treated with RBD-S19-TAT loaded with siRNA against the AR gene (grey) or the CSK gene (white). After treatment, the total RNA was extracted and subjected to cDNA synthesis, and the relative expression of the CSK gene **a** or the AR gene **b** was determined by RT–qPCR using the comparative CT method based on the expression level of the housekeeping gene RPL37A. The relative expression levels of CSK **a** and AR **b** mRNA were normalized by the mRNA expression using lipofection of the negative-control siRNA against AR **a** or CSK **b**, respectively. N = 3; means ± SD; *p < 0.05, **p < 0.01

To confirm whether siRNA taken up into cells causes gene knockdown, we used two siRNAs targeting endogenous CSK and AR genes. Each siRNA/RBD-S19-TAT protein complex was added to HeLa cells or prostate cancer-derived LNCaP cells that highly expressed AR, and the amount of CSK or AR mRNA was quantified by RT–qPCR. As expected, a decrease in CSK mRNA (Fig. 3a) or AR mRNA (Fig. 3b) was observed when the corresponding siRNA was bound to RBDwt-S19-TAT protein, while no decrease was observed when RBDwt-TAT protein was used (Fig. 3ab), indicating that S19 promotes the cytoplasmic delivery of siRNA.

**Fig. 4:**
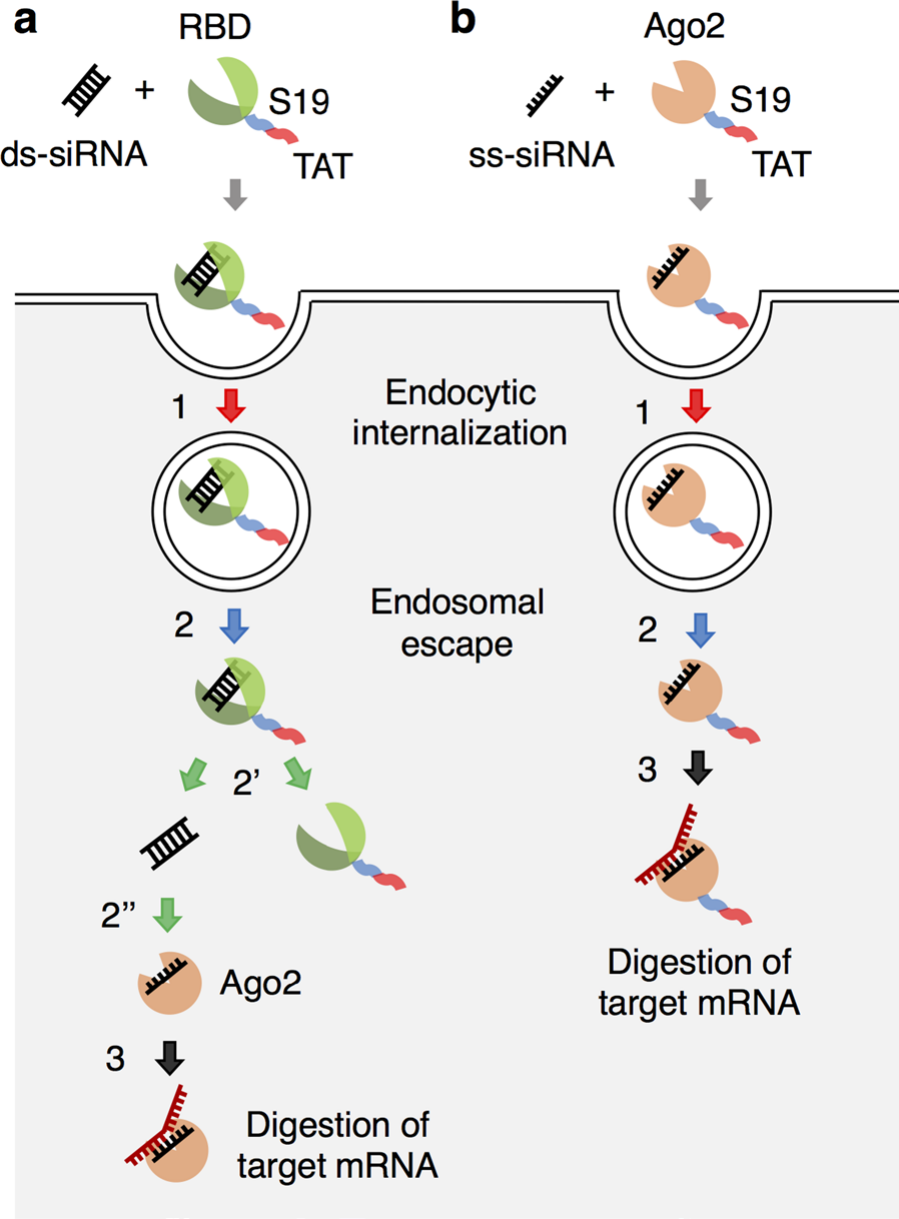
Schematic representation of the cytoplasmic delivery of siRNA in this study. By using **a** RBD or **b** Ago2 as the carrier protein fused with the membrane-penetrating peptides S19 and TAT, siRNA/protein complexes can be incorporated into cells by macropinocytosis (Step 1) and escape from endosomes (Step 2). Although **a** siRNA must dissociate from RBD-S19-TAT (Step 2') and reassociate with Ago2 (Step 2") to form RISC (Step 3), **b** the TAT-S19-Ago2/siRNA complex is expected to act immediately as RISC (Step 3)

Unexpectedly, however, no decrease in CSK mRNA was observed when the S19-TAT-fused RBDmut protein with higher affinity was used (Fig. 3a), despite the higher uptake of siRNA/RBDmut-S19-TAT compared to siRNA/RBDwt-S19-TAT (Fig. 1c). The reason is speculated to be as follows. After the RBD/siRNA complex is delivered to the cytoplasm, siRNA must dissociate from RBD and bind to Ago2 to form RISC (Fig. 4a). The binding between siRNA and S19-TAT-fused RBDmut protein might be too strong, so that siRNA cannot dissociate from RBD in the cytoplasm and then cannot be loaded into RISC. To overcome this problem, we next investigated whether it is possible to reliably induce RNAi after uptake into cells by fusing the S19 and TAT peptides with Ago2 instead of RBD for binding to siRNA (Fig. 4b). Although five chaperones are required for the in vitro reconstitution of human Ago2 and ds-siRNA [36], a high-concentration of single-stranded (ss)-siRNA also binds to Ago2 without chaperones [37–40]. Thus, we used ss-siRNA in the subsequent experiments.

### Cytoplasmic delivery of the siRNA/Ago2 complex using the TAT-S19 peptide

**Fig. 5:**
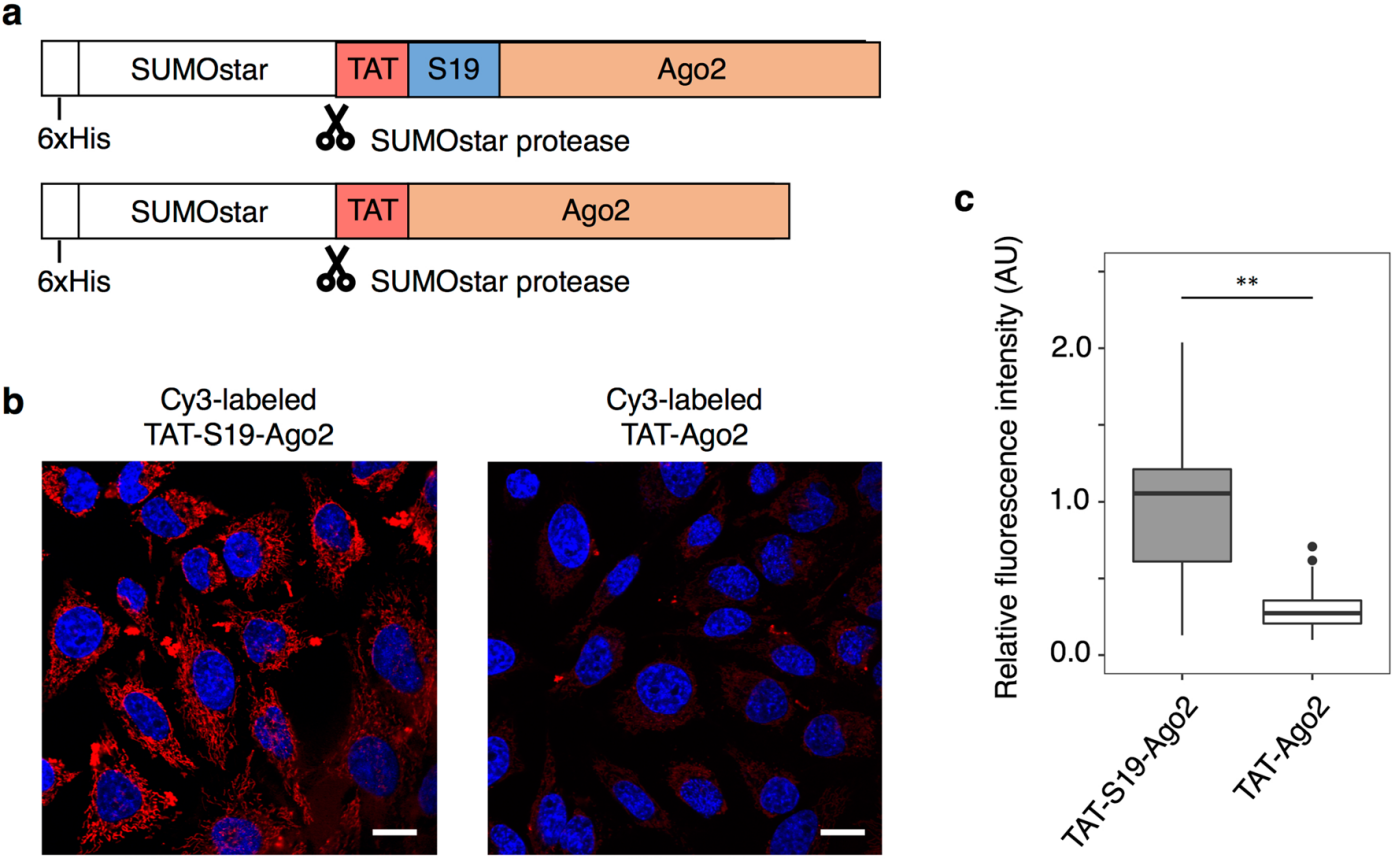
Intracellular delivery of Ago2 fusion proteins. **a** DNA constructs for Ago2 fusion proteins. **b** Confocal images of HeLa cells treated with 100 nM of Cy3-labelled Ago2 fusion proteins (red) for 1 h. Nuclei were stained with Hoechst 33,342 (blue). Scale bars, 20 μm. **c** ROI was taken for each cell and the average fluorescence intensity of Cy3 was quantified. N = 40 or 54; means ± SD; **p < 0.01

We constructed plasmids based on the pFastBac1 vector containing the polyhedrin promoter (P_PH_) for the insect cell expression of Ago2 fusion proteins, in which the TAT-S19 or TAT peptide as well as the 6 × His-SUMOstar-tag [41] were fused to the N-terminus of Ago2 (Fig. 5a), because the C-terminal amino acid of Ago2 has been shown to be involved in binding to siRNA [42]. Recently, we investigated the appropriate linking position and order of the S19 and TAT peptides to a cargo protein and found that both the previous C-terminal S19-TAT tag [16] and the N-terminal TAT-S19 tag promote the cytoplasmic delivery of the fusion protein [43]. SUMOstar-tag is a mutant SUMO-tag that is not cleaved by the endogenous protease in insect cells [41]. We expressed the Ago2 fusion proteins in Sf9 insect cells, purified them using a 6 × His-tag, and cleaved their SUMOstar-tag using SUMOstar protease (Additional file 1: Fig. S4).

Next, we examined the intracellular uptake of the Ago2 fusion proteins. The cysteine residues of the purified Ago2 proteins were fluorescently labelled using Cy3-maleimide, and the Cy3-labelled Ago2 fusion proteins were added to HeLa cells and observed with LSCM. As a result, the Cy3 fluorescence of TAT-S19-Ago2 in cells was higher than that of TAT-Ago2 (Fig. 5bc), indicating that S19 promoted the intracellular uptake of TAT-fused Ago2.

**Fig. 6:**
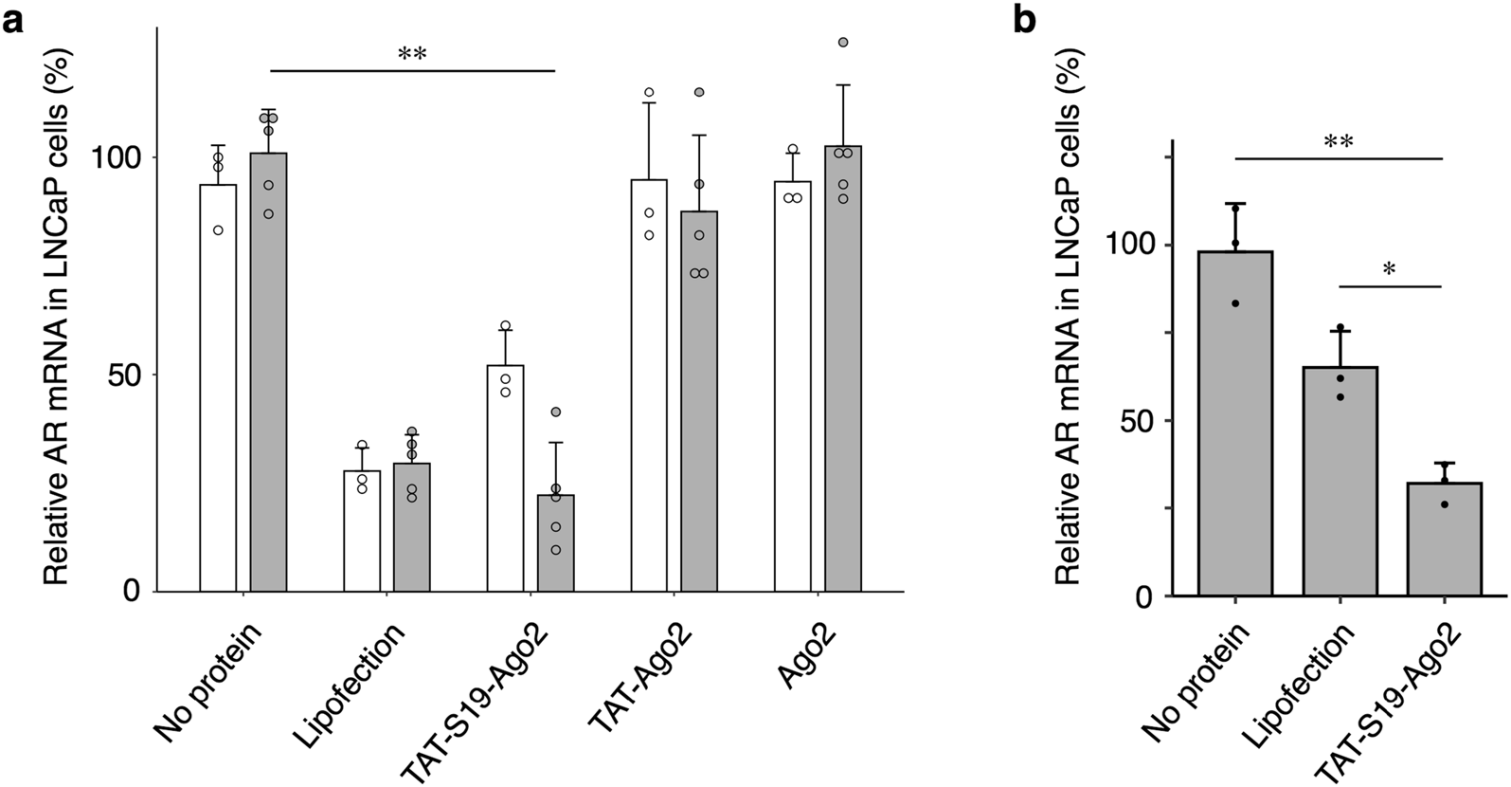
Gene knockdown with the TAT-S19-Ago2 protein/siRNA complex. LNCaP cells were treated with 200 nM (grey) or 100 nM (white) of the protein/siRNA complex. After 24 h **a** or 6 h **b**, the total RNA was extracted and used for cDNA synthesis, and the relative expression of the AR gene was determined by RT–qPCR using the comparative CT method based on the expression level of the housekeeping gene RPL37A. The relative expression levels of AR mRNA were normalized to the mRNA expression of free protein (200 nM siRNA). N = 5 or 3; means ± SD; *p < 0.05, **p < 0.001

Finally, we confirmed whether the siRNA complex with the TAT-S19-Ago2 protein was delivered to the cytoplasm and caused gene knockdown. Here, we used a chemically modified ss-siRNA (Additional file 1: Table S2) because ss-siRNA is more easily degraded than ds-siRNA. We mixed ss-siRNA targeting the AR gene with each Ago2 fusion protein, added them to LNCaP cells for 24 h, and verified the gene knockdown activity by RT–qPCR. As shown in Fig. 6a, a reduction in AR mRNA was observed for TAT-S19-Ago2, while no decrease in the mRNA was observed when TAT-Ago2 or commercially available Ago2 protein was used. These data clearly show that S19 promoted the cytoplasmic delivery of the Ago2/siRNA complex. The knockdown efficiency obtained with the TAT-S19-Ago2/siRNA complex after 24 h was comparable to that obtained with commercially available lipofectamine and siRNA (Fig. 6a, Lipofection). On the other hand, when the time of addition to cells was shortened from 24 to 6 h, the knockdown efficiency obtained by lipofection was reduced, but the knockdown efficiency obtained by using TAT-S19-Ago2 was not reduced (Fig. 6b). These results suggest that the delivery of the TAT-S19-Ago2/siRNA complex induced faster knockdown than traditional siRNA lipofection, probably due to the shorter time required for RISC formation in the cytoplasm.

## Discussion

In the present study, we prepared fusion proteins in which the membrane penetration-enhancing peptide S19 and the classical cell-penetrating peptide TAT were tandemly fused with siRNA-binding proteins (RBD or Ago2) and found that the efficiencies of siRNA delivery and knockdown obtained using RBD-S19-TAT or TAT-S19-Ago2 were higher than those using RBD-TAT or TAT-Ago2, respectively. The mechanism by which S19 promotes cytoplasmic delivery of TAT-fused proteins was presumed to be that S19 promotes TAT dimerization by parallel β-sheet formation between two S19-TAT peptides in the late endosomes (LEs), because (i) the negatively charged phospholipid in the LE membrane is known to interact with the TAT peptide electrostatically [44, 45], (ii) the dimerization of the TAT peptide has been reported to promote endosomal escape from LEs [14, 46, 47], (iii) the S19-TAT peptide has a β-structure in the presence of liposomes mimicking LEs [16], and (iv) the Ala-scanning mutagenesis of S19 indicated that amino-acid residues with high β-sheet forming propensities in S19 are important for the delivery of S19-TAT-fused proteins [43].

As mentioned in the background section, RBD has thus far been used as the carrier protein for siRNA delivery by multiple research groups. For example, Danielson et al. fused TAT to RBD, as in this study, but improved the affinity with siRNA by linking two RBDs forming a dimer with a polypeptide linker [31]. Furthermore, since the knockdown efficiency with this protein/siRNA complex alone was quite low, they co-added E5-TAT peptide in *trans* as an endosome-disrupting agent to promote endosomal escape for efficient knockdown [31]. Yang et al. fused the high-affinity RBDmut used in this study with EGFR-binding proteins for the delivery of siRNA into EGFR-expressing cells [32]. They also co-added an endosome-disrupting agent in *trans* to realize more efficient knockdown than that of lipofection [32]. In this study, by fusing the membrane penetration-enhancing peptide S19 with TAT in the wild-type RBDwt, we succeeded in achieving knockdown efficiency comparable to that of lipofection without the addition of any endosome-disrupting agent. On the other hand, in contrast to the results of Yang et al. [32], when we used the high-affinity RBDmut, the knockdown activity was lower than that of the wild-type RBDwt. (Fig. 3). We speculate that the reason is as follows. Since the membrane penetration-enhancing peptide S19 is thought to promote dimer formation [16, 43], the affinity of RBDmut-S19-TAT fusion protein with siRNA may have been further improved by this dimerization or by the interaction between cationic TAT and anionic siRNA, resulting in less dissociation in the cytoplasm and consequent difficulty in RISC formation.

As far as we know, there are no examples of using Ago2 alone as a carrier protein for siRNA. Li et al. recently reported that the codelivery of Ago2 and siRNA with transfection reagents, causes RNAi with higher efficiency than the delivery of siRNA alone [48]. They also proposed that one of the major barriers to RNAi-based therapy is the formation of a complex of siRNA and Ago2 after siRNA is released from a carrier in the cytoplasm, as shown in Fig. 4. The major advantage of using the TAT-S19-Ago2 protein as a carrier of siRNA is that rapid and efficient RNAi can be expected. Indeed, RBDwt-S19-TAT showed lower RNAi efficiency than lipofection (Fig. 3b), whereas TAT-S19-Ago2 showed better RNAi efficiency than lipofection (Fig. 6b). Therefore, we concluded that changing the carrier protein from RBD to Ago2 is effective. Although Li et al. also reported that the Ago2 complex with ds-siRNA was more potent than that with ss-siRNA with low stability [48], we achieved efficient knockdown by using the complex between the TAT-S19-Ago2 protein and ss-siRNA with standard chemical modifications (Additional file 1: Table S2; 5’-phosphate [39], 2’-methoxy [48–51] and 2’-fluoro [49, 52]).

In addition, the use of the TAT-S19-Ago2/siRNA complex may have the benefit of reducing off-target effects and cytotoxicity. It has been suggested that the delivery of siRNA causes off-target effects due to nonspecific competition with the endogenous small RNA pathway [53] and that the overexpression of siRNA suppresses the natural function of Ago2 and leads to cytotoxicity [54]. To address this problem, several researchers suggested that the coexpression of Ago2 during siRNA delivery induced not only an increase in RNAi efficiency but also a reduction in off-target effects and cytotoxicity [48, 54, 55]. The same effects can be expected in the codelivery of the TAT-S19-Ago2/siRNA complex used in this study, and we will verify these effects in the future.

One of the drawbacks of using Ago2 as the carrier protein is the difficulty of preparing Ago2 in large quantities thus far because Ago2 protein is extremely unstable and has problems such as being adsorbed on beads too strongly to elute during affinity purification. However, Tsuboyama et al. [56] recently discovered Hero proteins that stabilize Ago2 and other intrinsically disordered proteins, which are expected to promote the efficient elution and mass preparation of the TAT-S19-Ago2 protein for future therapeutic applications.

Recently, Ide et al. succeeded the hepatocyte-selective delivery of protein-therapeutics by using our membrane penetration-enhancing peptide S28 derived from syncytin 1 and a fragment antibody against the asialoglycoprotein receptor [57]. Since the TAT peptide used in this study has low cell-selectivity, it is desirable to realize the tissue- or cell-specific delivery of siRNA by fusing ligands/antibodies with receptors/markers that are specifically expressed in target cells instead of fusing TAT to Ago2 together with membrane penetration-enhancing peptides. Although there have been many reports of the direct conjugation of ligands/antibodies to siRNA, the problem of low endosomal escape efficiency remained [19, 20, 58, 59]. The addition of membrane penetration-enhancing peptides and Ago2, that is, the use of Ago2, which is a fusion of a ligand/antibody and a membrane penetration-enhancing peptide, as an siRNA carrier, can be expected to lead to more efficient cell-specific delivery and RNAi.

## Conclusion

In this study, we applied the membrane penetration-enhancing peptide S19 derived from human syncytin 1 to the cytoplasmic delivery of siRNA. We prepared fusion proteins in which the S19 and TAT peptides were fused to the RNA-binding proteins RBD or Ago2 as carrier proteins and investigated the cytoplasmic delivery of the siRNA/protein complex and the knockdown efficiency of the target genes. As expected, the siRNA/protein complex was incorporated into cells by TAT-mediated cell uptake, and the S19 peptide promoted the efficiency of cytoplasmic delivery and gene knockdown. In particular, it was suggested that the use of Ago2 as a carrier protein enables rapid and efficient knockdown. We believe that such protein-based methods have the potential to develop novel small delivery vehicles with heightened tissue permeability for RNAi-based therapy.

## Materials and methods

### Plasmid construction

All primers used in this study were purchased from Eurofins Genomics (Additional file 1: Table S1). All plasmids were constructed by the cloning method SLIC [34] using the following PCR products with overlap sequences.

For the construction of plasmids encoding 6 × His-SUMO-RBDmut-S19-TAT and 6 × His-SUMO-RBDmut-TAT, each insert was amplified from the RBDmut gene [32] synthesized by Eurofins Genomics with PrimeSTAR Max DNA polymerase (Takara) using the primers SUMO-RBD-F and RBD-S19-R or SUMO-RBD-F and RBD-TAT-R, respectively. Each vector was amplified from a pET vector encoding 6 × His-SUMO-eGFP-S19-TAT [16] using the primers S19-F and SUMO-R or TAT-F and SUMO-R, respectively. The resulting PCR products for the inserts and vectors were digested with DpnI (New England Biolabs) and purified using the QIAquick PCR extraction kit (Qiagen) before SLIC.

The above plasmids were used as templates for the construction of plasmids encoding 6 × His-SUMO-RBDwt-S19-TAT and 6 × His-SUMO-RBDwt-TAT by PCR using the primers K15N-R16G-F and K15N-R16G-R.

For the construction of plasmids encoding 6 × His-SUMOstar-TAT-S19-Ago2, the Ago2 gene was amplified from a cDNA clone purchased from DNAFORM using the primers Ago2-F and Ago2-R. The SUMOstar gene [41] was constructed by mutagenesis of the SUMO gene: a pET vector encoding 6 × His-SUMO-TAT-S19-eGFP [43] was amplified using the primers R66T-F and R66T-R, and the resulting plasmid was amplified using the primers R73E-F and R73E-R. The resulting vector was amplified using the primers pET15-F and Ago2-S19-R and joined to the insert Ago2 gene described above using SLIC. From the resulting vector, the 6 × His-SUMOstar-TAT-S19-Ago2 gene was amplified as the insert using the primers pFastBac-His-F and pFastBac-Ago2-R and cloned into the pFastBac1 vector (Thermo Fisher Scientific) amplified using the primers pFastBac-F and pFastBac-R by SLIC, resulting in pFastBac-6 × His-SUMOstar-TAT-S19-Ago2.

pFastBac-6 × His-SUMOstar-TAT-Ago2 was constructed from the above plasmid by PCR using the primers Ago2-F and Ago2-TAT-R and self-ligation by SLIC.

### Protein expression and purification

RBD fusion proteins were expressed in *E. coli* BL21(DE3)-RIPL cells (Agilent Technologies). For protein expression, *E. coli* cells transformed with each plasmid encoding the corresponding protein were grown in LB medium containing 100 µg/mL ampicillin at 37 °C until the OD_600_ reached 0.4–0.5. Then, 1 mM (final) isopropyl β-D-1-thiogalactopyranoside was added, and the cells were further cultivated at 25 °C for 24 h. Each protein was purified from the soluble fraction using cOmplete His-Tag Purification Resin (Sigma–Aldrich), as previously described [16]. Briefly, phosphate-buffered saline (PBS, Nacalai Tesque) supplemented with 500 mM NaCl and 10 mM imidazole and 0.1% Tween 20, PBS supplemented with 500 mM NaCl and 300 mM imidazole, and PBS supplemented with 500 mM NaCl were used as the wash, elution, and exchange buffers, respectively. The N-terminal 6 × His-SUMO tag was digested with Ulp1 at 4 °C overnight and removed using cOmplete His-Tag Purification Resin. The expression and purification of each protein were confirmed using 12.5% SDS–PAGE with Coomassie brilliant blue staining.

Ago2 fusion proteins were expressed using the Baculovirus Expression System (Thermo Fisher Scientific). Sf9 insect cells (5.0 × 10^4^ cells/mL) were infected with virus for 96 h, harvested, and washed in PBS. Cells were pelleted by centrifugation at 2,000 × g for 5 min and resuspended in 5 mL of lysis buffer [1 × TBS (Tris-buffered saline), 0.5 M NaCl, 1 mM TCEP, 1% (v/v) NP40] and 50 μL of ProteaseGuard EDTA-Free Protease Inhibitor Cocktail (Thermo Fisher Scientific). The cells were lysed with a Dounce tissue grinder. The cell lysate was centrifuged at 15,000 × g for 30 min, and the supernatant was collected as a soluble fraction. Each protein was purified using cOmplete His-Tag Purification Resin as described above. The N-terminal 6 × His-SUMOstar tag was digested with SUMOstar protease (LifeSensors) at 4 °C overnight and removed using cOmplete His-Tag Purification Resin. The expression and purification of each protein were confirmed using 10% SDS–PAGE with Coomassie brilliant blue staining.

### siRNA

The siRNAs used in this study were purchased from Gene Design. Their sequences (ds-siRNA for CSK [60] and AR [61], and ss-siRNA for AR [62]) and modifications are given in Additional file 1: Table S2.

### Electrophoretic mobility shift assay

Cy3-labelled siRNA (100 nM) was mixed with 0–800 nM purified RBD fusion protein in binding buffer [20 mM Tris–HCl (pH 7.6), 100 mM NaCl, 1 mM EDTA, 0.02% (v/v) TritonX-100, 2 mM dithiothreitol] at 4 °C for 1 h. The samples were then analysed by electrophoresis, where 5 × TBE sample buffer [90 mM Tris–HCl (pH 7.6), 90 mM boric acid, 2 mM EDTA, 15% Ficoll type 400, 0.02% xylene cyanol] was diluted to 1 × TBE in the binding reaction, and then 12.5 μL of the sample was applied to a 6% TBE gel and electrophoresed at a constant voltage of 100 V for 60 min in 0.5 × TBE buffer. Cy3 fluorescence was detected using ChemiDoc Touch MP (BioRad).

### siRNA stability assay in serum

Cy3-labelled siRNA (100 nM) was mixed with 200 nM purified RBD fusion protein in 1 × PBS buffer at 4 °C for 1 h. Then, this solution was mixed with foetal bovine serum (Nichirei Biosciences), and the mixture was incubated at 37 °C for 3, 24 and 48 h. After 6% TBE gel electrophoresis, the Cy3 fluorescence was detected using ChemiDoc Touch MP to confirm the remaining Cy3-siRNA. The residual rate of siRNA was calculated from the band intensity.

### Cell lines and cell culture

The human cervical cancer cell line HeLa (RIKEN Cell Bank) and human prostate cancer cell line LNCaP (RIKEN Cell Bank) were maintained in Dulbecco's modified Eagle's medium (DMEM) (Nacalai Tesque) or RPMI-1640 (Nacalai Tesque) with 10% (v/v) foetal bovine serum (Nichirei Biosciences) and 1% (v/v) penicillin–streptomycin (Life Technologies) and incubated at 37 °C and 5% CO_2_ in static culture.

The insect cell line sf9 (Thermo Fisher Scientific) was maintained in Grace’s Insect Medium (Thermo Fisher Scientific) with 10% (v/v) foetal bovine serum and 1% (v/v) penicillin–streptomycin (Life Technologies) and incubated at 27 °C.

### Fluorescent imaging

The cells were seeded in glass-bottom dishes (AGC Techno Glass) for 24 h before the experiments. Each RBD fusion protein (400 nM) and Cy3-labelled siRNA (100 nM) were mixed at a molar ratio of 4:1 (RBD:siRNA) and incubated at 4 °C for 30 min. A fluorescently labelled Ago2 fusion protein was obtained by mixing the Ago2 fusion protein and Cy3 maleimide (Lumiprobe) at 4 °C for 24 h. The sample medium was replaced by DMEM containing the RBD/Cy3-siRNA complex or Cy3-labelled Ago2 (100 nM) and Hoechst 33,342 at 1 μg/mL (Life Technologies). After 1 h of incubation, the cells were washed three times with PBS. The medium was replaced with DMEM without phenol red (Nacalai Tesque) containing 10% (v/v) foetal bovine serum and 1% (v/v) penicillin–streptomycin and observed by LSCM (FV10i-DOC, Olympus). Uptake via the endocytosis pathway was confirmed using various endocytosis inhibitors, as previously described [16].

### RT–qPCR

The 1.0 × 10^5^ HeLa cells or 5.0 × 10^4^ LNCaP cells were seeded in a 24-well plate 24 h before the experiments. RBD or Ago2 fusion proteins and siRNA were mixed at a molar ratio of 4:1 (RBD:siRNA) or 1:1 (Ago2:siRNA) and incubated at 4 °C for 30 min or 1 h, respectively. Native Ago2 protein purchased from SinoBiological was used as a control. The protein/siRNA complex (100–200 nM) was added to a 24-well plate. As a positive control, siRNA was transfected using Lipofectamine 2000 (Thermo Fisher Scientific). After a predetermined time (3, 6, 12 or 24 h), total cellular RNA was extracted using the RNeasy Plus Mini Kit (Qiagen). Then, the mRNA in the total RNA was converted to cDNA with M-MLV Reverse Transcriptase (Promega) using the Oligo dT primer (Additional file 1: Table S1). The resulting cDNA was amplified with a 2 × TB Green Premix *ExTaq* II (Tli RNaseH Plus) (Takara) using the primers CSK-sense and CSK-antisense, AR-sense and AR-antisense, or RPL37A-sense and RPL37A-antisense (Additional file 1: Table S1) on a LightCycler (Roche) and analysed using the comparative CT method.

## Supplementary Information

**Additional file 1: Fig. S1.** Expression and purification of RBD fusion proteins. **Fig. S2. ** siRNA stability assays. **Fig. S3.** Confocal images of HeLa cells treated with the complex of RBDmut fusion protein and Cy3-labelled siRNA. **Fig. S4.** Expression and purification of Ago2 fusion proteins. **Table S1.** Primer sequences used in this study. **Table S2.** siRNA sequences and modifications used in this study.

## Data Availability

All data generated or analysed during this study are included in the article and the supplementary information file.

## Abbreviations

Ago2: Argonaute 2
AR: Androgen receptor
CPP: Cell-penetrating peptide
CSK: C-terminal Src kinase
DMEM: Dulbecco's modified Eagle's medium
Ds: Double-stranded
EDTA: Ethylenediaminetetraacetic acid
GalNAc: N-acetylgalactosamine
GFP: Green fluorescent protein
LNP: Lipid nanoparticle
LSCM: Laser scanning confocal microscope
MβCD: Methyl-β-cyclodextrin
PBS: Phosphate-buffered saline
RBD: RNA-binding-domain
RISC: RNA-induced silencing complex
RNAi: RNA interference
RT–qPCR: Reverse transcription–quantitative polymerase chain reaction
SDS–PAGE: Sodium dodecyl sulfate–polyacrylamide gel electrophoresis
siRNA: Small interfering RNA
ss: Single-stranded
TBE: Tris-borate-EDTA
TBS: Tris-buffered saline
