# Supplementary material for: Cytoplasmic delivery of siRNA using human-derived membrane penetration-enhancing peptide
Source: J Nanobiotechnology. 2022 Oct 27;20:458. doi: 10.1186/s12951-022-01667-4 (PMC9615171; doi:10.1186/s12951-022-01667-4)
Supplement: Supplementary file 1 — Additional file 1: Fig. S1. Expression and purification of RBD fusion proteins. Fig. S2. siRNA stability assays. Fig. S3. Confocal images of HeLa cells treated with the complex of RBDmut fusion protein and Cy3-labelled siRNA. Fig. S4. Expression and purification of Ago2 fusion proteins. Table S1. Primer sequences used in this study. Table S2. siRNA sequences and modifications used in this study. [file 12951_2022_1667_MOESM1_ESM.pdf]

Supplementary Information for

Title

Cytoplasmic delivery of siRNA using human-derived membrane penetration-enhancing peptide

Authors

Momoko Nakamura, Kei Fujiwara, Nobuhide Doi

Affiliations

Department of Biosciences and Informatics, Keio University, 3-14-1 Hiyoshi, Yokohama 223-8522, Japan

Contents

Fig. S1: Expression and purification of RBD fusion proteins

Fig. S2: siRNA stability assays

Fig. S3: Confocal images of HeLa cells treated with the complex of RBDmut fusion protein and Cy3-labelled siRNA

Fig. S4. Expression and purification of Ago2 fusion proteins

Table S1: Primer sequences used in this study

Table S2: siRNA sequences and modifications used in this study

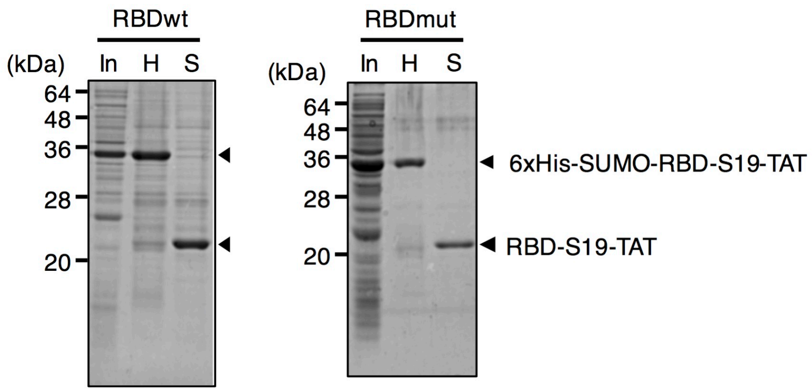

Fig. S1. Expression and purification of RBD fusion proteins. RBDwt-S19-TAT protein (left) and RBDmut-S19-TAT protein (right) with N-terminal 6xHis-SUMO tag were expressed in *E. coli* (lane 'In'; soluble fraction) and purified by affinity purification using 6xHis-tag (lane 'H'). Then the N-terminal SUMO-tag was cleaved with Ulp1 (lane 'S'). The samples were analysed by 12.5% SDS–PAGE with Coomassie brilliant blue staining.

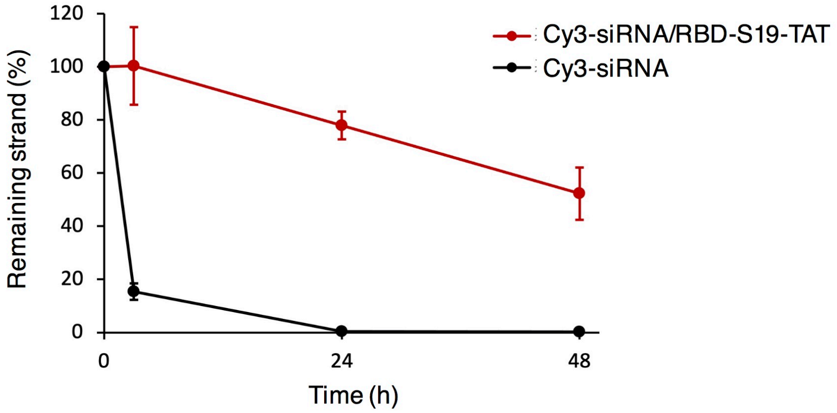

Fig. S2. siRNA stability assays. The Cy3-labelled siRNA (black line) or the Cy3-siRNA complex with the RBD-S19-TAT protein (red line) was exposed to bovine serum, and the Cy3 fluorescence of siRNA band was separated by native PAGE and quantified by an image analyzer. N=3; means ± SD.

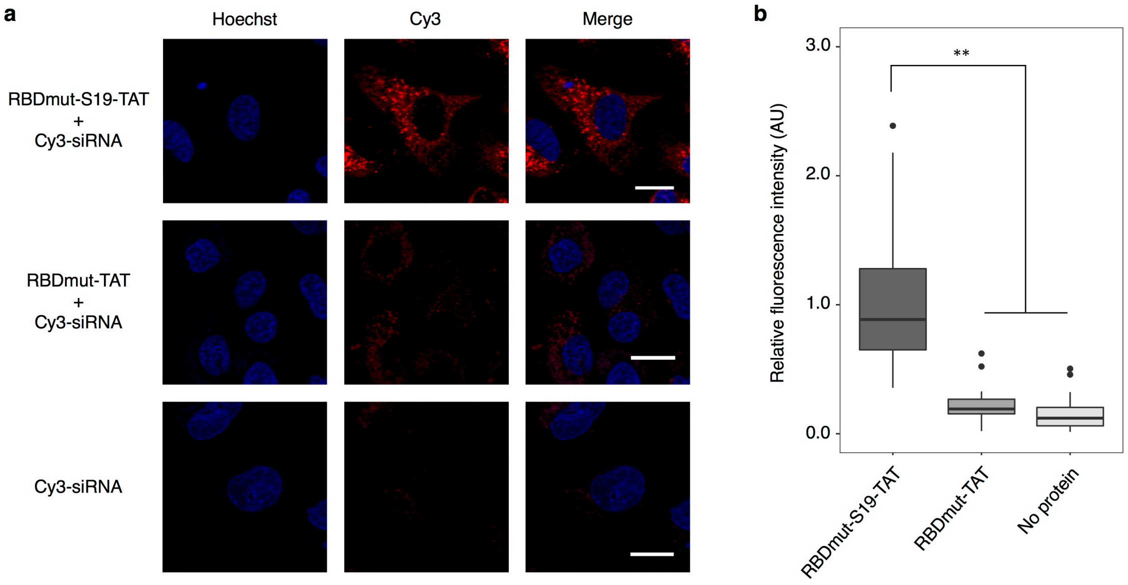

Fig. S3. Confocal images of HeLa cells treated with the complex of RBDmut fusion protein and Cy3-labelled siRNA. (**a**) RBDmut-S19-TAT or RBDmut-TAT protein (400 nM) was mixed with Cy3-siRNA (100 nM) at 4°C for 1 h for binding. HeLa cells were treated with the protein/Cy3-siRNA complex (red) for 1 h. Nuclei were stained with Hoechst 33342 (blue). Scale bars, 20 μm. (**b**) ROI was taken for each cell and the average fluorescence intensity of Cy3 was quantified. N = 52, 20 or 26; means ± SD; $^{**}p < 0.01$.

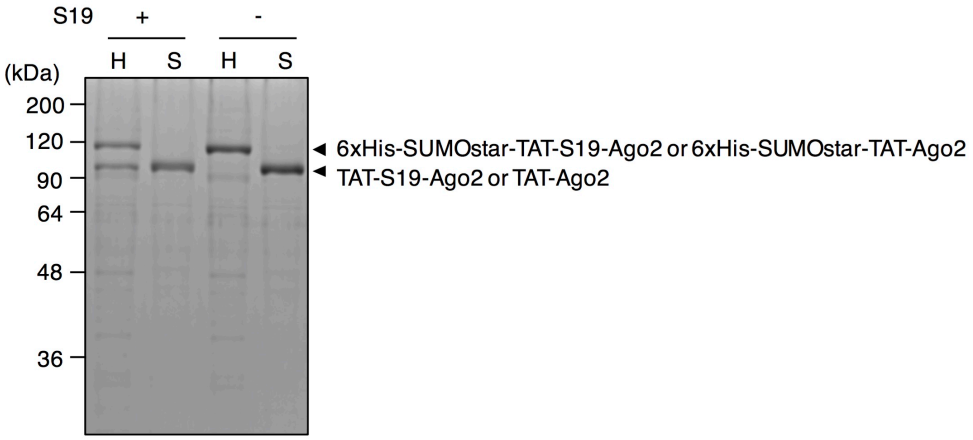

Fig. S4. Expression and purification of Ago2 fusion proteins. TAT-S19-Ago2 and TAT-Ago2 proteins with N-terminal 6xHis-SUMOstar tag were expressed in Sf9 insect cells and purified by affinity purification using 6xHis-tag (lane 'H'). Then the N-terminal SUMOstar-tag was cleaved with SUMOstar protease (lane 'S'). The samples were analysed by 10% SDS–PAGE with Coomassie brilliant blue staining.

Table S1. Primer sequences used in this study

Name	DNA sequence (5' to 3')
SUMO-RBD-F	GAACAGATTGGAGGTATGGAACGCGCGATTCA
RBD-S19-R	GCACCGATCACAAACGGTTCACTCTCTTTCTTGAAG
RBD-TAT-R	CTCTTTTTACGGCCGTATTCACTCTCTTTCTTGAAG
S19-F	CCGTTTGTGATCGGTGCC
TAT-F	TACGGCCGTAAAAAGAGA
SUMO-R	ACCTCCAATCTGTTCGC
K15N-R16G-F	CGTTCGCCGTTCGCTTGCTCACGTGTG
K15N-R16G-R	AGCGAACGGCGAACGTTGGGATGGCG
Ago2-F	ATGTACTCGGGAGCCGG
Ago2-R	AGCAAAGTACATGGTGCGCAG
R66T-F	TCCTTAACGTTCTTGTACGACGGTATTAGAATT
R66T-R	CAAGAACGTTAAGGAGTCCATTTCCTTACCCTG
R73E-F	GGTATTGAAATTCAAGCTGATCAG
R73E-R	TTGAATTTCAATACCGTCGTACAAG
pET15-F	GGATCCGGCTGCTATC
Ago2-S19-R	CGGCTCCCGAGTACATAATCCCGCCAATGCCAG
pFastBac-His-F	CATCGGGCGCATGGGTCATCAC
pFastBac-Ago2-R	CCGCATGCCTCGAGTCAAGCAAAGTACATGGTGCG
pFastBac-F	CTCGAGGCATGCGGTAC
pFastBac-R	GCGCCCGATGGTGGAC
Ago2-TAT-R	GGCTCCCGAGTACATACGTCTACGTTGGCGTC
Oligo dT	TTTTTTTTTTTTTTT
CSK-sense	CCTGAGGCCCTGAGAGAGAA
CSK-antisense	GATAAGGCACTCGCCCAAAG
AR-sense	GCCTTGCTCTCTAGCCTCAA
AR-antisense	GGTCGTCCACGTGTAAGTTG
RPL37A-sense	ATTGAAATCAGCCAGCACGC
RPF37A-antisense	GCAGGAACCACAGTGCCAGATCC

Table S2. siRNA sequences and modifications used in this study

Name	Strand	Sequence (5' to 3') [modification]
CSK-siRNA	Guide	[Cy3-]CUACCGCAUCAUGUACCAUtt
	Passenger	AUGGUACAUGAUGCGGUAGtt
AR-siRNA	Guide	GAUGAAGCUUCUGGGUGUCACUAUG
	Passenger	CAUAGUGACACCCAGAAGCUUCAUC
AR-ss-siRNA	Guide	p-mGfUmGfAmGfCmUfGmGfUmAfGmAfAmGfCmGfUmC<br>[p, 5'-phosphate; mN, 2'-methoxy; fN, 2'-fluoro]
